# Elabela gene therapy promotes angiogenesis after myocardial infarction

**DOI:** 10.1111/jcmm.16814

**Published:** 2021-07-21

**Authors:** Liangli Jin, Yang Pan, Quanyi Li, Jing Li, Zhi Wang

**Affiliations:** ^1^ Department of Cardiovascular Medicine Affiliated Nanjing Brain Hospital Nanjing Medical University Nanjing China

**Keywords:** adeno‐associated virus, angiogenesis, Elabela, gene therapy

## Abstract

This study was aimed at investigating whether Elabela (ELA) gene therapy can promote angiogenesis in the treatment of myocardial infarction (MI). The fusion expression plasmid pAAV‐3 × Flag/ELA‐32 was successfully constructed using molecular cloning technique. The model of acute MI was established by ligating the left anterior descending coronary artery in mice. Adeno‐associated virus serotype 9 (AAV9) was injected into the surrounding myocardium and tail vein immediately after the model was established. AAV was injected again from the tail vein one week later. Compared with the MI+PBS (control) group, the serum N‐terminal pro‐brain natriuretic peptide (NT‐proBNP) concentration, and the values of left ventricular end‐diastolic diameter (LVDd) and left ventricular end‐systolic diameter (LVDs) of the MI+AAV‐ELA (gene therapy) group were significantly decreased, while the value of left ventricular ejection fraction was significantly increased at 2 and 4 weeks after operation. Compared with the control group, the expression of CD105 and vWF and the percentage of CD31‐ and Ki67–co‐positive cells were significantly increased in the gene therapy group. Moreover, the expressions of apelin peptide jejunum (APJ) receptor, vascular endothelial growth factor (VEGF), VEGFR2, Jagged1 and Notch3 in the heart tissue around the infarction were up‐regulated in mice with gene therapy. The results suggest that ELA activates VEFG/VEGFR2 and Jagged1/Notch3 pathways through APJ to promote angiogenesis after myocardial infarction. ELA gene therapy may be used in the treatment of ischaemic cardiomyopathy in future.

## 1 INTRODUCTION

With the ageing of the world population, the incidence rate of coronary heart disease is increasing. Coronary heart disease has become a global public health problem. Among these concerns, myocardial infarction is a critical cardiovascular disease threatening human health, and it is the main cause of sudden death and heart failure. Active and effective reperfusion therapy, including thrombolytic therapy, percutaneous transluminal angioplasty and coronary artery bypass grafting, can save the dying myocardium and reduce myocardial ischaemic necrosis. However, these reperfusion methods are non‐physiological.^1^ Therapeutic angiogenesis provides a new therapeutic strategy for myocardial infarction. The basis for therapeutic angiogenesis is to improve myocardial perfusion and cardiac function by promoting the formation of neovascularization in ischaemic areas of the heart.^2^

Elabela (apela or toddler) is a newly discovered non‐coding RNA transcription gene. This gene can transcribe and translate to produce a 55‐amino acid secretory hormone. After enzyme cleavage, it forms a 32‐amino acid mature polypeptide (ELA‐32). Through the study of zebrafish, a model animal, it was found that the hormone acts on the apelin peptide jejunum (APJ) receptor as apelin and plays an important role in the development of the embryonic cardiovascular system. ELA appears earlier than apelin in the process of embryonic development, and it is the earliest ligand acting with the APJ receptor. The embryos with ELA gene or APJ gene knockout had a similar phenotype of cardiac developmental malformation, suggesting that ELA plays an important role in embryonic cardiovascular development.^3^, ^4^ A basic research showed that ELA‐32 could not only promote the formation of vascular tubular structures in human umbilical vein endothelial cells in vitro but also improve the cardiac function of mice with acute myocardial infarction (MI).^5^, ^6^ This study further explored whether ELA‐32 gene therapy can promote angiogenesis in the treatment of myocardial infarction.

## 2 MATERIALS AND METHODS

### 2.1 Production and infection of adeno‐associated virus (AAV)

#### 2.1.1 Construction of AAV expression vector

Specific primers were designed according to the coding sequence of ELA‐32. The primer sequences were as follows: forward 5ʹ‐GACGCTAGC TTCATCACCGAGGAGAAATC‐3ʹ, reverse 5ʹ‐CCTGATATC GAATGCAGTGAGATACACCT‐3ʹ. PCR products with *Nhe*‐*I* and *EcoRV* restriction enzyme sites were purified and recovered. T4 ligase was used to ligate the target gene with the empty vector of ITR‐CMV‐PolyA‐ITR (Dorn) digested by *Nhe*‐*I* and *EcoRV* at 16℃. The positive clones were selected, and the constructed plasmid was extracted and amplified. The recombinant pAAV‐3 × Flag/ELA‐32 plasmid was identified by sequencing and enzyme digestion.

#### 2.1.2 Packaging of AAV serotype 9 (AAV9)

293T cells (ATCC) in good growth condition were seeded on 6‐well plates at a density of 4 × 10^5^ cells/well, cultured in an incubator at 37℃ in 5% CO_2_ and then transfected when 80%–90% confluence was reached. The 293T cells were transfected with the packaging plasmid pAAV, helper plasmid (Han Bio, Shanghai, China) and pAAV‐3 × Flag/ELA‐32 plasmid mixed in a certain proportion using the LipoFiter^TM^ transfection reagent (Han Bio, Shanghai, China). After 72 h of transfection, the cells were lysed and centrifuged at 4℃ at 53,000 R/min for 30 h. The virus was purified by iodixanol density gradient centrifugation. The collected virus was resuspended in phosphate‐buffered saline (PBS) and stored at −80℃. The copy number of virus genome was determined by quantitative PCR.

#### 2.1.3 Infection of 293T with AAV9

293T cells in good growth condition were counted and then seeded on a 6‐well plate with 1 × 10^6^ cells/well. The infection was carried out when the cells showed 70%–80% confluence. The fresh culture medium was replaced, and the adeno‐associated virus solution diluted by PBS (multiplicity of infection, MOI = 10^6^) was added. After 24 h of incubation in a 5% CO2 incubator at 37℃, the fresh culture medium was replaced. After 48 h, the cells were collected for protein extraction and Western blotting.

### 2.2 Establishment of myocardial infarction model in mice

Eight‐week‐old male SPF‐grade C57BL/6 mice were purchased from Model Animal Research Center of Nanjing University. Mice were raised in 22℃ ± 2℃ environment, with food and water available *ad libitum*. After anaesthesia, the skin and subcutaneous tissue were cut along the anterior midline of the neck of the mice, the tracheal intubation was inserted, and the ventilator was connected to control the breathing (90–100 breaths/min, tidal volume 0.4–0.5 ml). The chest was cut longitudinally to expose the heart. The left anterior descending coronary artery was ligated with a 7–0 suture about 1.5 mm below its junction, and the skin was sutured gradually. After waking up, the mice were extubated and observed for 15 min. Animal experiment was approved by the Committee on Ethics in the Care and Use of Laboratory Animals of Nanjing Medical University.

### 2.3 Grouping of experimental animals

The experiment was divided into the following groups: (1) the sham group, wherein the heart of mouse was exposed without ligation; (2) the MI+PBS group, wherein 100‐µl PBS was injected into the tail vein and the borders of the infarction immediately after successful modelling and another 100 μl PBS was injected into the tail vein one week later in the same MI mouse; and (3) the MI+AAV‐ELA group, wherein 100 μl 6 × 10^11^ genome copies/animal AAV‐ELA was injected into the tail vein and the borders of the infarction immediately after successful modelling and another 100 μl 6 × 10^11^ genome copies/animal AAV‐ELA was injected into the tail vein one week later in the same MI mouse. In our present study, the injected AAV dose was calculated according to the animal bodyweight (3 × 10^13^ genome copies/kilogram/animal).^7^ The average weight of the 8‐week‐old mice was 20 g; thus, the viral copy number used was determined as 6 × 10^11^ genome copies/animal.

### 2.4 Echocardiography examination

Echocardiography was performed at 2 and 4 weeks after model preparation, as previously described. The mice were anaesthetized with 1.5%–2% isoflurane and fixed in supine position to maintain spontaneous breathing. The VEVO 770 35‐MHz high‐frequency probe was placed in the left chest of mice to obtain satisfactory two‐dimensional images of the long axis and short axis of the left ventricle beside the sternum. The heart rate was measured. The M‐mode ultrasonic electrocardiogram was obtained by placing the M‐mode sampling line perpendicular to the interventricular septum and the posterior wall of the left ventricle at the level of the papillary muscle in the short‐axis section. Left ventricular end‐diastolic diameter (LVDd) and left ventricular end‐systolic diameter (LVDs) were measured. After storing the images, the left ventricular ejection fraction (LVEF) was analysed offline. Calculation formula was LVEF = (LVDd–LVDs)/LVDd × 100%.

### 2.5 Detection of N‐terminal pro‐brain natriuretic peptide (NT‐proBNP)

Blood samples of mice were collected before modelling and 2 weeks and 4 weeks after intervention. Serum endogenous NT‐proBNP (Cloud‐Clone Corp) was measured by ELISA according to the manufacturer's instructions.

### 2.6 Immunohistochemical and immunofluorescence test

The dried paraffin sections were dewaxed with xylene and hydrated with gradient ethanol. After antigen repair, the paraffin sections were incubated with 3% H_2_O_2_ for 10 min at room temperature and washed with PBS 3 times. The tissue sections were incubated overnight at 4°C with primary antibody (CD105: 1:100; vWF: 1:100; Abcam). On the next day, reagent A and reagent B were added sequentially, and finally, chromogenic solution DAB (Maxim) was added. The slices were dehydrated and sealed with neutral gum. A field of vision was randomly selected and photographed with phase‐contrast microscope.

For double‐fluorescent staining, the paraffin sections of mouse heart tissue were incubated at room temperature and soaked in 0.4% Triton X‐100 for 10 min. After blocking with 5% sheep serum at 37℃ for 10 min, the sections were incubated overnight at 4℃ with primary antibodies (CD31:1:100; ki67:1:50; Abcam). The fluorescent‐labelled secondary antibody (Abcam) was added at the next day and incubated at 37℃ for 1 h in the dark. After washing with PBS and sealing with 90% glycerine, the sections were imaged on a laser scanning confocal microscope.

### 2.7 Reverse Transcription‐Polymerase Chain Reaction (RT‐PCR)

Total RNA was extracted from tissues using TRIzol reagent and cDNA synthesized using the HiScript RT Reagent Kit (Vazyme, China). The primer sequences for transfected gene 3 × Flag/ELA‐32 and endogenous housekeeping gene β‐actin were as follows: 3 × Flag/ELA‐32: forward 5′‐TTGGGTCTTCTTCATCTTC‐3′, reverse 5′‐TAGCACGCACTGTAGTTTCT‐3′; and β‐actin: forward 5′‐TAGCACGCACTGTAGTTTCT‐3′, reverse 5′‐TCTTCCTTCCGACCTTTT‐3′. Real‐time PCR was run in 96‐well plates using a SYBR qPCR Master Mix (Vazyme, China) according to the instruction of the manufacturer. All reactions were conducted using the ABI 7300 Real Time PCR System (Applied Biosystems). Analysis of relative gene expression was performed using the comparative C*t* method.

### 2.8 Western blot

The cultured cells or heart tissue in each group was lysed in RIPA buffer. The protein concentration was detected using a BCA protein quantitative detection kit. The protein sample (30 μg) was loaded on a 10% SDS‐PAGE gel. Electrophoresis was performed, followed by electrophoretic transfer of the proteins to a PVDF membrane. After blocking with 5% skim milk, the membrane was incubated with TBS‐T–diluted primary antibody [rabbit anti‐mouse Flag, VEGF, VEGFR2, Jagged1 (Cell Signaling Technology), Notch3 (ProteinTech Group) and GAPDH (Abcam)] at 4℃ overnight. The next day, the membrane was washed three times with TBS‐T and then incubated with horseradish peroxidase–labelled secondary antibody (sheep anti‐rabbit IgG; Abcam) at room temperature for 1 h. The grey value of each band was quantified using Quantity One software.

### 2.9 Statistical analyses

Data were analysed using GraphPad Prism 8 statistical software. Data of normal distribution were presented as mean ± SEM. A non‐paired *t* test was used in the comparison of fewer than three groups. Statistical differences among more than three groups were evaluated by one‐way ANOVA and Tukey's test. *p* < 0.05 was considered statistically significant.

## 3 RESULTS

### 3.1 Successful construction of the expression plasmid pAAV‐3×Flag/ELA‐32

**FIGURE 1:**
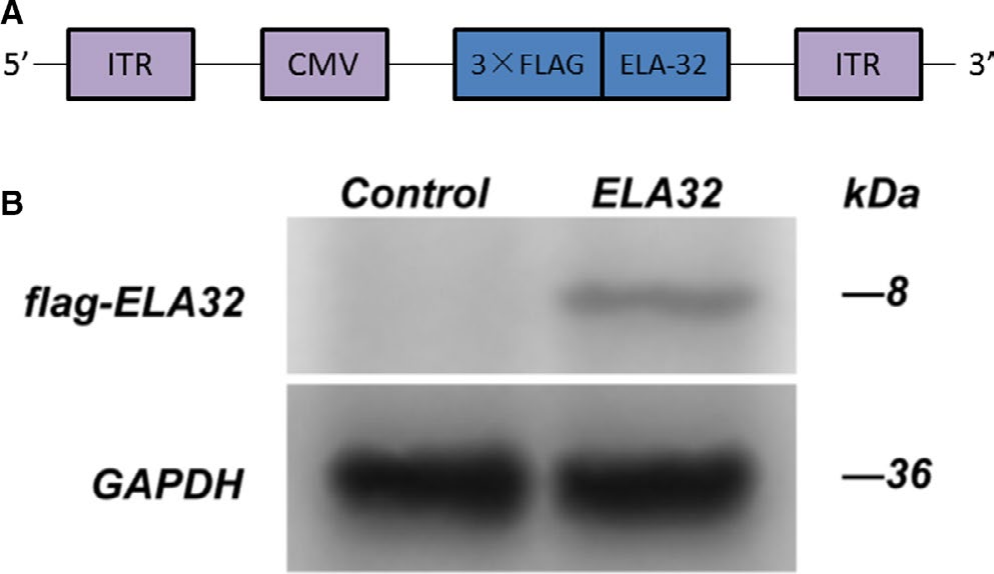
Construction of adeno‐associated virus (AAV) expression vector and infection of 293T cells with AAV. The fusion expression plasmid pAAV‐3 × Flag/ELA‐32 was successfully constructed by molecular cloning technique. (A) shows the scheme of AAV construct of 3 × Flag/ELA‐32 expression vector. 293T cells were able to express the recombinant ELA protein after infection with AAV (B)

The fusion expression plasmid pAAV‐3 × Flag/ELA‐32 was successfully constructed by molecular cloning technique (Figure 1A). The correct tail‐to‐head orientation of the coding sequence of ELA in pAAV‐3 × Flag/ELA‐32 was confirmed by sequencing analysis. The protein of 293T cells infected by AAV was extracted 48 h after infection. Western blotting showed that 293T cells expressed the 3 × Flag/ELA‐32 fusion protein (Figure 1B).

### 3.2 The expression of 3×Flag/ELA‐32 after AAV intervention in vivo

**FIGURE 2:**
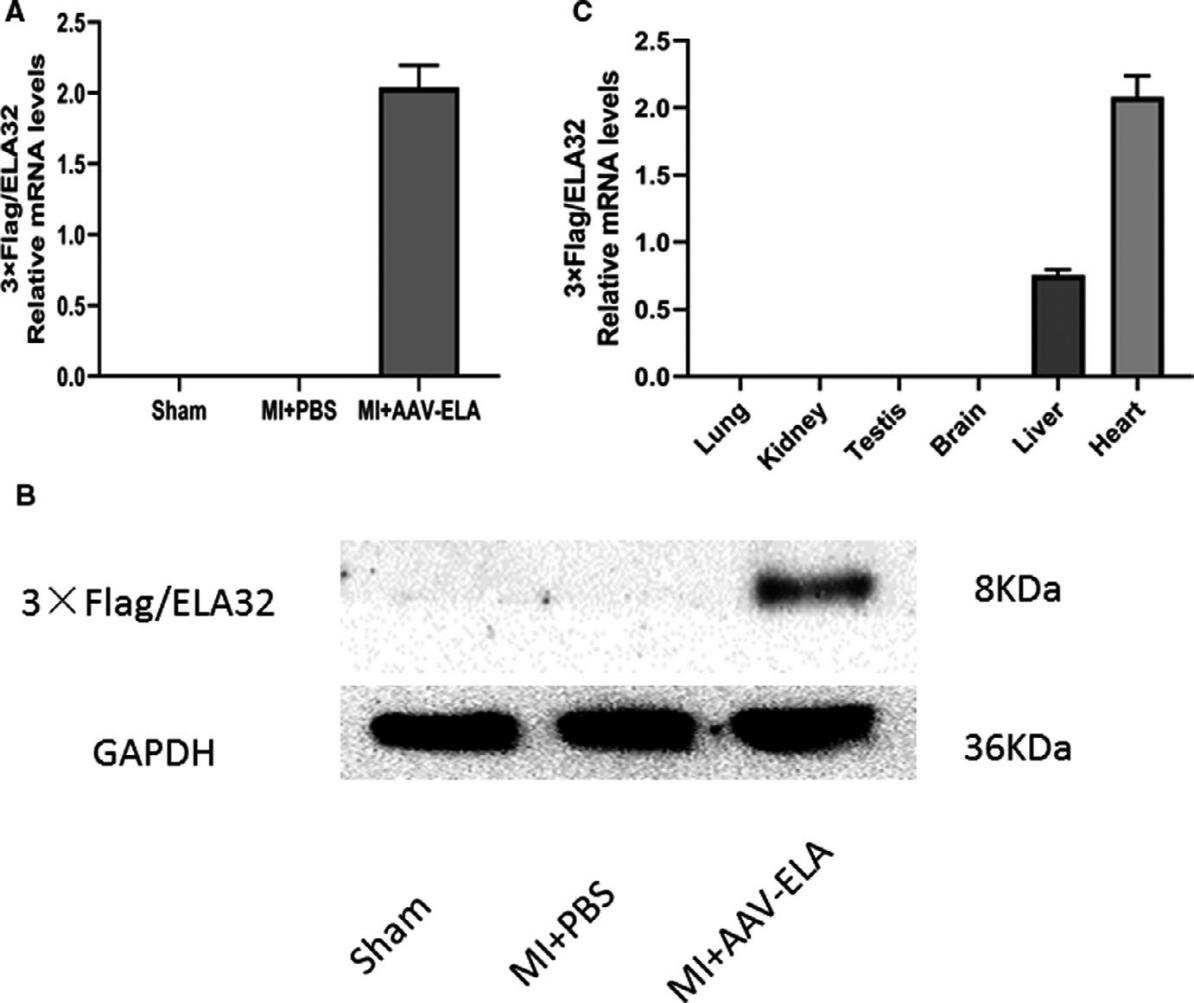
Expression of AAV‐delivered 3 × Flag/ELA in vivo. RT‐PCR (A) and Western bolt (B) results showed that 3 × Flag/ELA was only expressed in the heart of MI mice intervened by AAV‐3 × Flag/ELA injection (*n* = 5) at the fourth week after the establishment of the MI model. The 3 × Flag/ELA was not detected in the heart of mice in MI +PBS (*n* = 5) or sham (*n* = 5) groups. RT‐PCR analysis showed that the expression of 3 × Flag/ELA mRNA was strongest in the heart with mild expression found in the liver and there was no expression in lung, kidney, brain and testis of MI mice 4 weeks after AAV9 injection (C)

At the fourth week after the establishment of the MI model and AAV intervention, total RNA and protein were extracted from heart tissue of experimental mice for RT‐PCR (Figure 2A) and Western blot (Figure 2B), respectively. The results showed that 3 × Flag/ELA was expressed only in the hearts of MI mice intervened by AAV‐3 × Flag/ELA injection. Further, 3 × Flag/ELA was not detected in the hearts of mice in the MI +PBS or sham group. In addition, total RNA was extracted from the heart, kidney, liver, brain, lung and testis tissue of MI mice 4 weeks after AAV9 injection. RT‐PCR analysis showed that the expression of 3 × Flag/ELA mRNA was strongest in the heart with mild expression found in the liver and there was no expression in lung, kidney, brain and testis (Figure 2C).

### 3.3 ELA gene therapy improves cardiac function in mice with myocardial infarction

**FIGURE 3:**
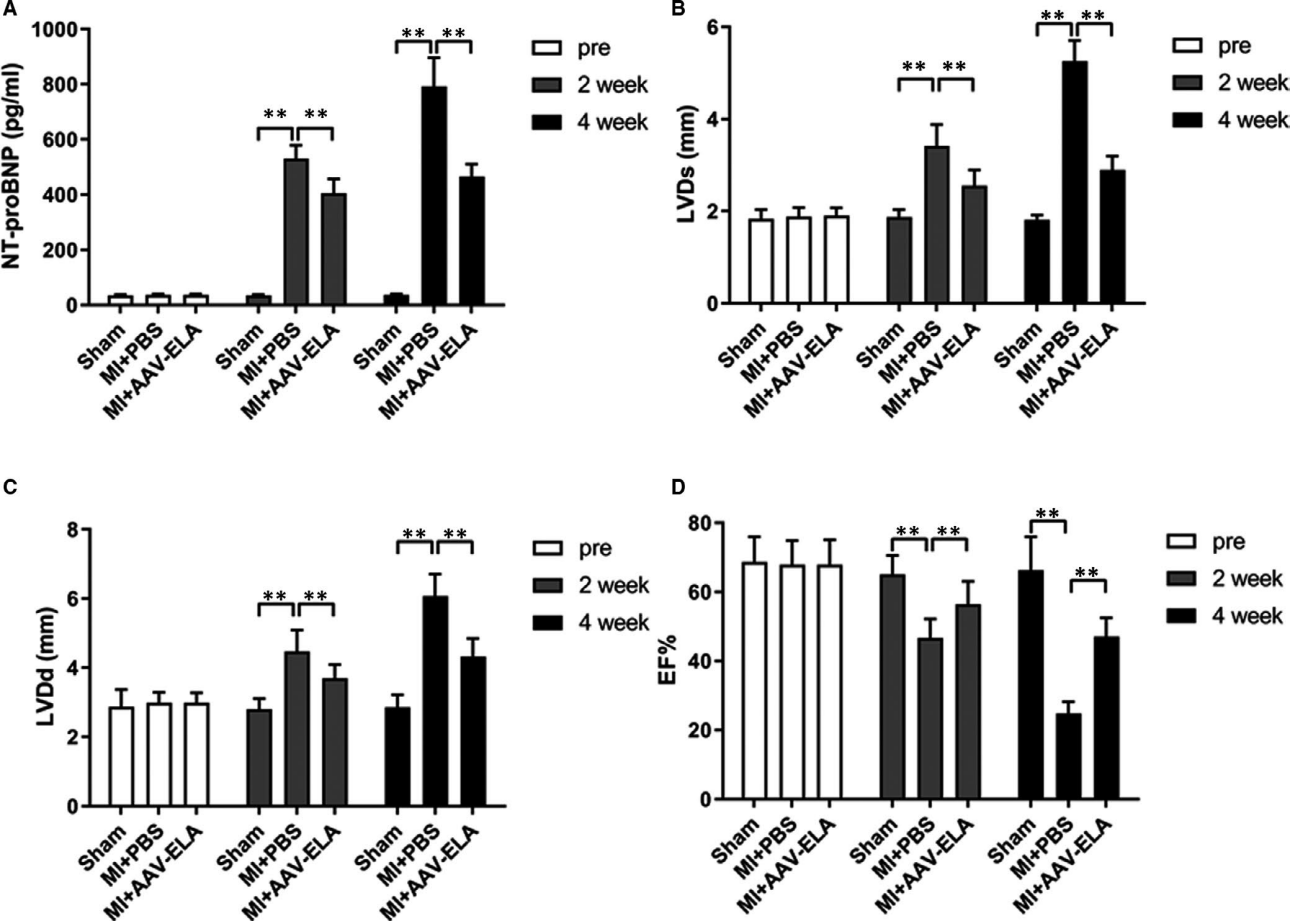
ELA gene therapy improves cardiac function in mice with myocardial infarction (MI). AAV was injected into the surrounding myocardium and tail vein immediately after the model was established. Then, AAV was injected again from the tail vein one week later. The N‐terminal pro‐brain natriuretic peptide (NT‐proBNP) content (A) in serum of mice from the indicated group (each group, *n* = 5) was detected at the time of modelling and at 2 and 4 weeks after operation. Left ventricle end‐systolic diameter (LVDs) (B) left ventricle end‐diastolic diameter (LVDd) (C) and left ventricular ejection fraction (LVEF) (D) were detected at indicated time. Values are mean ± SEM. **p < 0.01, compared with the MI+PBS group

As shown in Figure 3, there was no significant difference in values of LVDd (Figure 3C), LVDs (Figure 3B) and LVEF (Figure 3D) and serum levels of NT‐proBNP (Figure 3A) before operation among the groups. At 2 weeks after MI, compared with the sham group, the MI+PBS group showed significantly higher serum levels of NT‐proBNP and the values of LVDd and LVDs and a significantly lower value of LVEF; the difference increased further at 4 weeks after myocardial infarction. Compared with the MI+PBS group, the MI+AAV‐ELA group showed significantly decreased serum levels of NT‐proBNP and the values of LVDd and LVDs and a significantly increased value of LVEF at 2 and 4 weeks after MI.

### 3.4 ELA gene therapy promotes angiogenesis in mice with myocardial infarction

**FIGURE 4:**
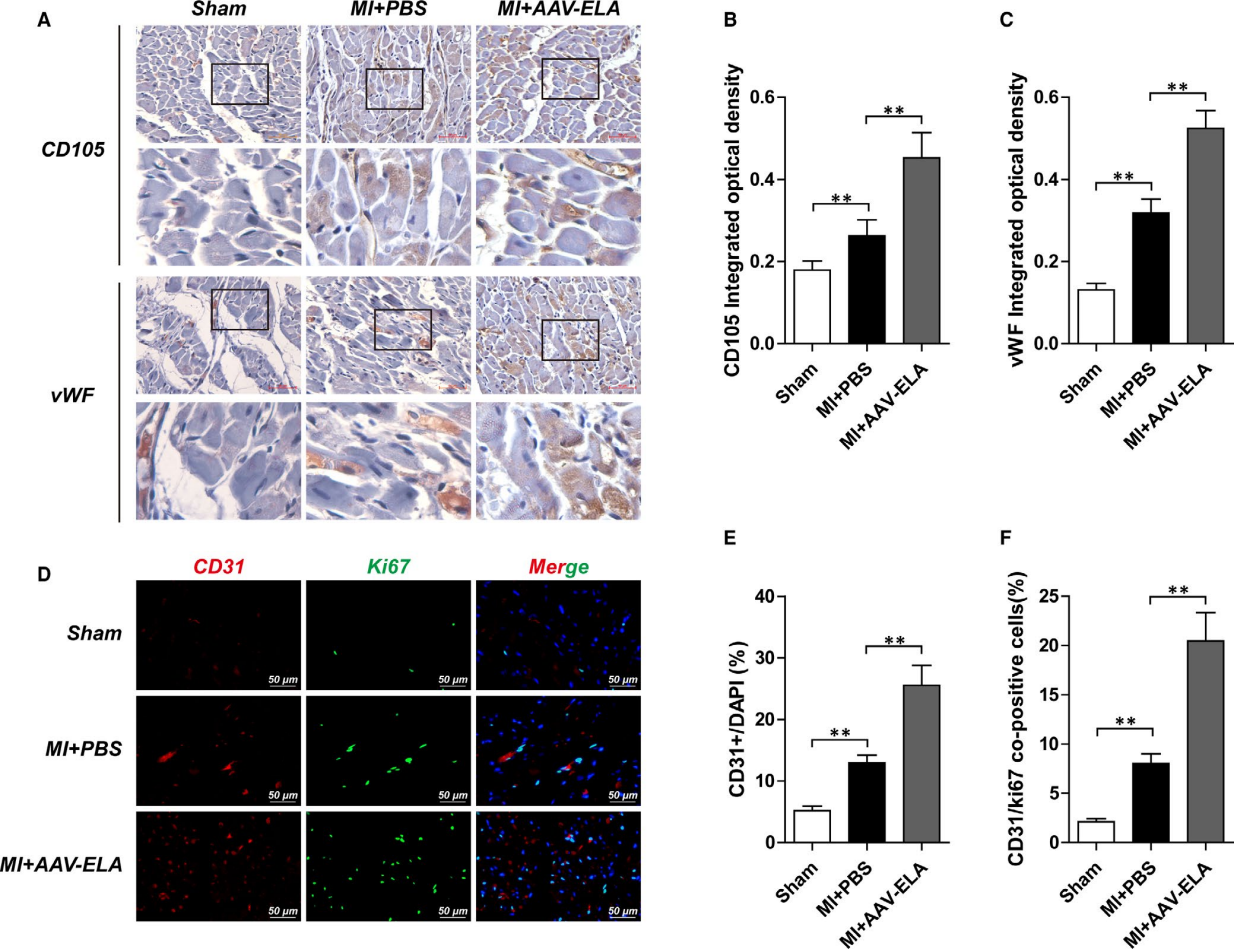
ELA gene therapy promotes angiogenesis in a mouse model of myocardial infarction. AAV was injected into the surrounding myocardium and tail vein immediately after the model was established. Then, AAV was injected again from the tail vein one week later. The expression of CD105 (A and B) and vWF (A and C) in heart was detected at 4 weeks after operation. The enlarged images are located at the bottom of the corresponding picture (black box). These show that CD105 or vWF staining is brown. (D) shows representative images of Ki67 proliferation marker (green), CD31 (red), and DAPI nuclear (blue) staining in heart, respectively. (E) reports the CD31+/DAPI ratios in each group (*n* = 5), while the percentage of CD31/Ki67–co‐positive cells are indicated in F. Values are mean ± SEM. **p < 0.01, compared with the MI+PBS group

At the fourth week after intervention, the hearts of the three groups of mice were taken out for immunostaining examination. The expression of CD105 and vWF (Figure 4A‐C) and the number of CD31‐ and Ki67 (Figure 4D‐F)–co‐positive cells near the area of infarction were higher in the MI+PBS group than in the sham group. Compared with the MI+PBS group, the MI+AAV‐ELA group showed significantly increased expression of CD105 and vWF and the percentage of CD31‐ and Ki67–co‐positive cells near the area of infarction.

### 3.5 ELA gene therapy increases VEGF/VEFGR2 and Jagged1/Notch3 expression in mice with myocardial infarction

**FIGURE 5:**
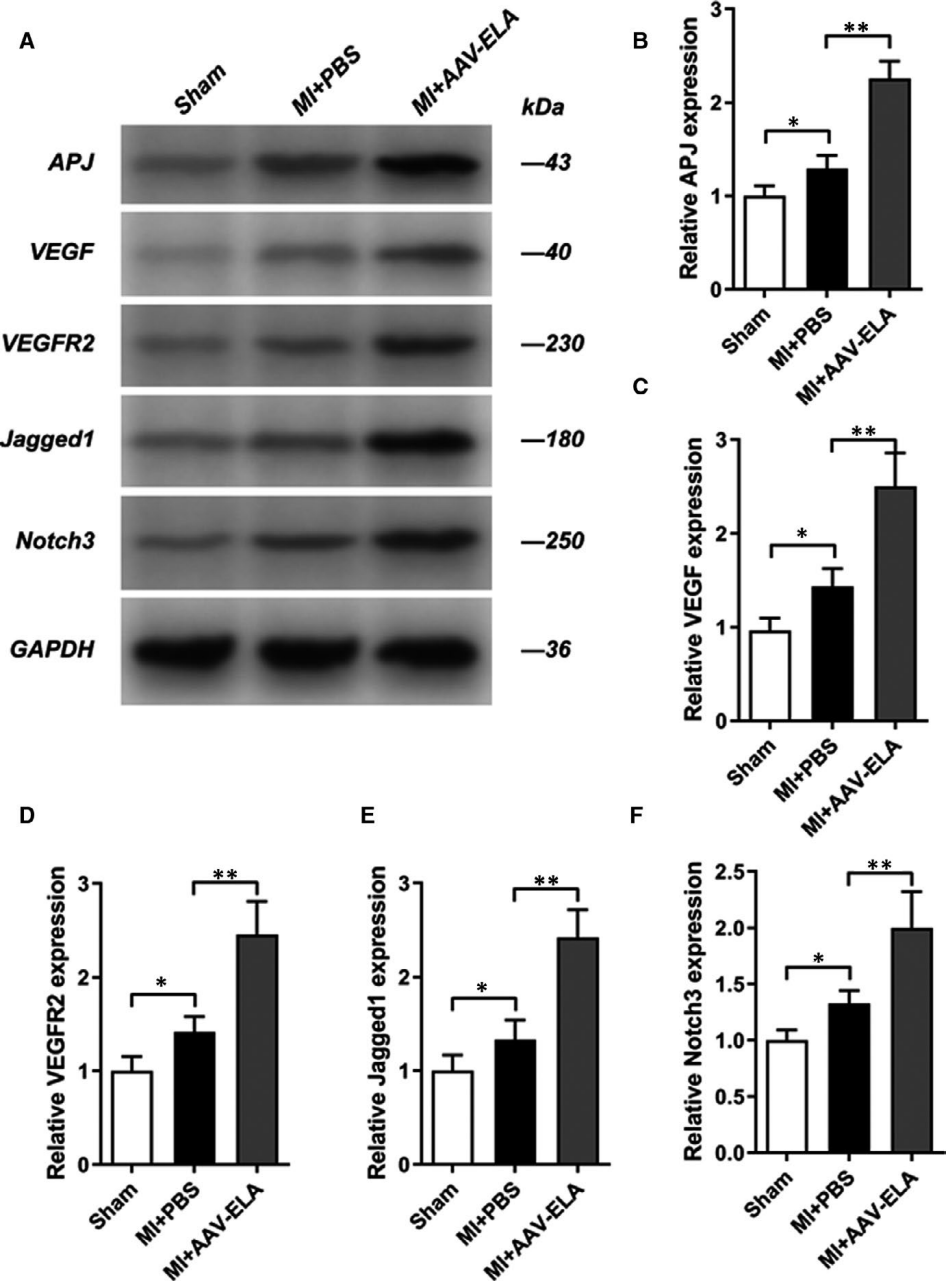
ELA gene therapy increases VEGF/VEFGR2 and Jagged1/Notch3 expression in mice with myocardial infarction. AAV was injected into the surrounding myocardium and tail vein immediately after the model was established. Then, AAV was injected again from the tail vein one week later. The expression of apelin peptide jejunum (APJ) receptor (A and B), vascular endothelial growth factor (VEGF, A and C), VEGFR2 (A and D), Jagged1 (A and E) and Notch3 (A and F) from the indicated group (each group, *n* = 5) was detected by Western blot assay at 4 weeks after operation. Values are mean ± SEM. *p < 0.05, compared with the sham group;**p < 0.01, compared with the MI+PBS group

At the fourth week after the intervention, the hearts of the three groups of mice were taken out to extract the total protein for Western blotting analysis (Figure 5A). In MI+PBS and MI+AAV‐ELA groups, the myocardial tissues around the infarction were extracted. The expression levels of the ELA receptor APJ (Figure 5B), VEGF (Figure 5C) and its receptor VEGFR2 (Figure 5D) and Jagged1 and its receptor Notch3 were detected. The expression of APJ, VEGF, VEGFR2, Jagged1 (Figure 5E) and Notch 3 (Figure 5F) was higher in the MI+PBS group than in the sham group. After ELA gene therapy, compared with the MI+PBS group, the expressions of APJ, VEGF, VEGFR2, Jagged1 and Notch3 were significantly up‐regulated in the MI+AAV‐ELA group.

## 4 DISCUSSION

Ischaemic cardiomyopathy is among the main causes of heart failure. In addition to drug therapy, percutaneous coronary revascularization and surgical coronary artery bypass grafting are also the main treatment methods. However, drug treatment has side effects, and minimally invasive intervention and surgery also have complications, such as in‐stent restenosis and coronary bridge occlusion.^8^ Therefore, cardiologists have been trying to treat ischaemic cardiomyopathy with new technologies and methods in recent years.

Stem cell transplantation has seen new progress in the treatment of ischaemic cardiomyopathy in recent years, and good results have been achieved in animal experiments. Researchers in France also reported the application of embryonic stem cells in the treatment of patients with end‐stage heart failure.^9^ However, issues such as ethical and oncogenic controversy limit the clinical application.^10^ Gene therapy is a hot spot in the treatment of ischaemic cardiomyopathy. Plasmids carrying genes encoding antioxidant, endothelial nitric oxide synthase, mitogen protein kinase, hepatocyte growth factor and vascular endothelial growth factor were transfected into animals via an adenovirus, and biological effects, such as heart protection and angiogenesis, were observed.^11^

The apelin/APJ system is involved in the regulation of the vascular diameter during angiogenesis in apelin‐knockout mice.^12^ The animal model of myocardial ischaemia also showed that apelin could up‐regulate the expression of phosphorylated endothelial nitric oxide synthase and vascular endothelial factor (VEGF) in ischaemic myocardium.^13^ The biological effects of apelin on mitosis and angiogenesis of endothelial cells suggest that apelin has a potential role in therapeutic angiogenesis.^14^ ELA is a newly discovered APJ ligand and is the earliest endogenous ligand to bind the APJ receptor in the process of development. The molecular structure shows that apelin and ELA are basic amino acids with the same equipotential point and that both of them are acting ligands of APJ. It is suggested that ELA and apelin have some biological commonalities. For example, both of them can induce human umbilical vein endothelial cells to form a vascular‐like tubular structure *in vitro* and both have biological effects, such as enhancing myocardial contractility and maintaining body fluid balance.^15^, ^16^, ^17^

Recently, preliminary studies have shown that exogenous administration of recombinant ELA can improve the cardiac function of mice with acute myocardial infarction and of rats with pressure overload heart failure.^6^, ^18^ However, due to the short half‐life of ELA, it needs to be continuously administered by a subcutaneous osmotic pump, and thus, some researchers are trying to use ELA gene therapy to achieve the purpose of long‐term expression of ELA *in vivo*.

Currently, AAV is commonly used in gene therapy. Previous studies have shown that different serotypes of AAV have different tissue affinities, and AAV9 has a strong affinity for the heart. Unlike other serotypes, the target gene carried by AAV9 can be persistently expressed in host tissue for up to a year.^19^, ^20^ A study by Zincarelli C *et al*. injected different serotypes of AAV carrying luciferase into the tail vein of mice. Luciferase expression was observed as early as 7 days after AAV9 injection. At day 100, luciferase expression was strongest in the heart and liver and then declined over 9 months.^21^ Schreiber CA *et al*. observed in animal experiments that the ELA transfected into hypertensive rats by AAV could significantly delay the occurrence of hypertension symptoms and that the experimental rats could still maintain the integrity of glomerular structure and inhibit renal fibrosis after 3 months of high‐salt diet.^22^ These results indicate that after AAV introduces the target gene in the body, somatic cells can continuously transcribe and express the target protein.

In this study, the AAV‐ELA plasmid was successfully constructed by gene recombination technology. After injected into mice via the tail vein and local intra‐myocardial injection, the cardiac function of mice with myocardial infarction was significantly improved, and the angiogenesis around the infarction was promoted. In our previous study, we continuously injected exogenous recombinant ELA in mice with MI by osmotic pump. We found that ELA inhibited myocardial fibrosis and apoptosis of myocardial and kidney cells and improved the heart and kidney function of mice with MI.^6^ Considering the multiple effects of ELA on the cardiovascular system, we speculate that cardiac function improvement is related to promoting angiogenesis, inhibiting myocardial fibrosis and apoptosis of myocardial cells.

We further observed that the expression of VEGF and VEGFR2 in the myocardial infarction control group was slightly up‐regulated, while that in the Ela gene therapy group was significantly up‐regulated. VEGF is a highly specific vascular endothelial growth factor, which can promote vascular permeability and endothelial cell migration, proliferation and angiogenesis. However, it also mediates angiogenesis in tumour growth, metastasis, diabetic retinopathy, myocardial ischaemia and other pathological states.^23^ VEGFR belongs to the receptor tyrosine protein kinase family and has five isomers. VEGFR2 is mainly expressed by endothelial cells and endothelial progenitor cells. The biological function of VEGF is mainly exercised by its interaction with VEGFR2. VEGF was expressed at low levels in the physiological state. In acute myocardial ischaemia, VEGF secretion could be enhanced by the stimulation of mechanical tension and local inflammatory factors.^24^, ^25^ Previous studies have shown that exogenous apelin can induce endothelial progenitor cells homing to ischaemic myocardium and promote angiogenesis.^13^ Combined with the results of this study, we speculate that ELA gene therapy up‐regulates VEGF/VEGFR2 in infarcted myocardium, which may be associated with promoting endothelial progenitor cells homing to ischaemic myocardium and promoting angiogenesis.

In this study, the expression of CD105 and vWF was significantly increased after ELA gene therapy. Immunofluorescence showed that the proportion of CD31/Ki67 double‐positive cells in the adjacent infarcted myocardium increased after ELA gene therapy. The results suggest increased density of neovascularization in the area adjacent to the infarcted myocardium. Western blotting results also showed that the expression of Jagged1/Notch3 in infarcted myocardium was increased after ELA gene therapy. Notch signalling pathway is a highly conserved signalling pathway widely existing in vertebrates and invertebrates. It regulates the differentiation and development of cells, tissues and organs through the interaction between adjacent cells.^26^, ^27^ In 1995, the ligand that can activate the Notch signalling pathway was cloned and named Jagged1. Jagged1/Notch system is related to heart development, and Jagged1 gene mutation can cause congenital heart disease.^28^ Notch3 gene mutation can reduce the diameter of the cerebral artery and microvessel density.^29^ In recent years, more and more attention has been paid to the role of Jagged1/Notch3 system in angiogenesis, particularly in tumour angiogenesis.^30^ Li L et al. found that apelin‐13 can mediate the proliferation of vascular smooth muscle cells, while ERK blocker can inhibit the activation of Jagged1/Notch3 pathway induced by apelin‐13.^31^ Recently, Chen *et al*. have found that apelin‐13/APJ can promote the growth and proliferation of colon cancer cells by activating Notch3 pathway and can inhibit the growth of tumour cells regardless of inhibiting APJ or Notch3 pathway.^32^ Based on this study, we also preliminarily observed that ELA‐32, a novel APJ ligand, can also up‐regulate Jagged1/Notch3 expression. It is speculated that there is a crosstalk between ELA/APJ pathway and Jagged1/Notch3 pathway, and the underlying mechanism needs to be further studied.

Several limitations of our study warrant discussion. First, we currently lack imaging technology to evaluate myocardial angiogenesis *in vivo*. Second, because of the significant effect of angiogenesis and the improvement of cardiac function at 4 weeks after AAV9 injection, we concluded the animal experiment at 4 weeks. Whether or not AAV9 gene therapy has an off‐target effect in the long term needs to be studied in the future.

## 5 CONCLUSIONS

In summary, our results suggest that ELA activates VEFG/VEGFR2 and Jagged1/Notch3 pathways through APJ to promote angiogenesis after myocardial infarction. ELA gene therapy may be used in the treatment of ischaemic cardiomyopathy in future.

## CONFLICTS OF INTEREST

The authors declare no any conflicts of interest in this work.

## AUTHOR CONTRIBUTION

**Liangli Jin:** Data curation (equal); Investigation (lead); Methodology (equal); Project administration (equal). **Yang Pan:** Data curation (equal); Methodology (equal); Project administration (equal); Writing‐original draft (equal). **Quanyi Li:** Conceptualization (equal); Data curation (equal); Methodology (equal); Project administration (equal). **Jing Li:** Data curation (equal); Investigation (equal); Methodology (equal). **Zhi Wang:** Conceptualization (lead); Investigation (lead); Methodology (equal); Project administration (lead); Writing‐original draft (lead); Writing‐review & editing (equal).

## Data Availability

All data generated or analysed during this study are included in this article.
